# Cardiogenic Shock Due to Progressive Heart Failure—Clinical Characteristics and Outcomes Compared to Other Aetiologies

**DOI:** 10.3390/biomedicines13081856

**Published:** 2025-07-30

**Authors:** Dominik Krupka, Michał Fułek, Julia Drewniowska, Kamila Florek, Mateusz Milewski, Michał Nnoli, Katarzyna Grunwald, Adam Chełmoński, Karolina Karska, Kacper Cicirko, Katarzyna Mazur, Jakub Ptak, Mikołaj Błaziak, Robert Zymliński, Waldemar Goździk, Barbara Barteczko-Grajek, Maciej Bochenek, Roman Przybylski, Michał Zakliczyński, Mateusz Sokolski, Wiktor Kuliczkowski

**Affiliations:** 1 Student Scientific Club of Transplantology and Advanced Therapies of Heart Failure, Institute of Heart Diseases, Faculty of Medicine, Wroclaw Medical University, 50-368 Wroclaw, Poland; 2 Clinical Department of Diabetology, Hypertension and Internal Diseases, Institute of Internal Diseases, Faculty of Medicine, Wroclaw Medical University, Borowska 213, 50-556 Wroclaw, Poland; 3 Collegium Medicum, Jan Kochanowski University, 25-317 Kielce, Poland; 4 Jan Mikulicz Radecki University Hospital, Borowska 213, 50-556 Wroclaw, Polandm.blaziak@umw.edu.pl (M.B.); waldemar.gozdzik@umw.edu.pl (W.G.); barbara.barteczko-grajek@umw.edu.pl (B.B.-G.);; 5 Clinical Department of Cardiology, Institute of Heart Diseases, Faculty of Medicine, Wroclaw Medical University, Borowska 213, 50-556 Wroclaw, Poland; 6 Clinical Department of Intensive Cardiac Care, Institute of Heart Diseases, Faculty of Medicine, Wroclaw Medical University, Borowska 213, 50-556 Wroclaw, Poland; 7 Department of Anesthesiology and Intensive Therapy, Wroclaw Medical University, Borowska 213, 50-556 Wroclaw, Poland; 8 Clinical Department of Heart Transplantation and Mechanical Circulatory Support, Department of Cardiac Surgery and Heart Transplantation, Institute of Heart Diseases, Faculty of Medicine, Wroclaw Medical University, Borowska 213, 50-556 Wroclaw, Poland; 9 Department of Cardiac Surgery and Heart Transplantation, Institute of Heart Diseases, Faculty of Medicine, Wroclaw Medical University, Borowska 213, 50-556 Wroclaw, Poland

**Keywords:** cardiogenic shock, heart failure, myocarditis, in-hospital mortality

## Abstract

**Background:** The prevalence of cardiogenic shock (CS) resulting from the progression of heart failure (PHF) is increasing and remains associated with high mortality. This study aimed to compare the clinical characteristics and outcomes of patients who developed CS due to PHF versus those whose CS was caused by other aetiologies (non-PHF). **Methods:** We retrospectively analysed 280 patients admitted to a Polish tertiary care centre between January 2021 and April 2024. The cohort was divided into two groups: PHF (*n* = 84, 30%) and non-PHF (*n* = 196, 70%). **Results:** Compared to the non-PHF group, PHF patients more frequently had chronic kidney disease (30% vs. 15%, *p* < 0.01), and significant valvular disease (30% vs. 13%, *p* < 0.01). PHF patients exhibited significantly lower white blood cell counts (9.4 [6.9–16.4] vs. 13.3 [10.4–17.6], *p* < 0.01) and troponin T levels (188 [61–1392] vs. 10,921 [809–45,792], *p* < 0.01). In-hospital mortality was significantly lower among PHF patients (52% vs. 65%, *p* = 0.04). Although the overall use of mechanical circulatory support (MCS) did not differ between groups, significant differences in the types of MCS applied were observed (*p* < 0.01). Additionally, PHF patients underwent fewer coronary revascularisation procedures (15% vs. 70%, *p* < 0.01). **Conclusions:** Patients with PHF-related CS exhibit distinct clinical profiles and may experience lower in-hospital mortality when appropriately diagnosed and treated with a personalised approach. Further prospective, multicentre studies are warranted to optimize the management of this growing subgroup of CS patients.

## 1. Introduction

Cardiogenic shock (CS) is a critical condition characterised by reduced cardiac output, leading to inadequate tissue perfusion, multiorgan dysfunction, and, if left untreated, death [1]. The underlying mechanisms of CS vary widely and can develop either acutely—as in the case of acute myocardial infarction (AMI)—or progressively, for example, in patients with chronic decompensated heart failure (HF) [2]. Despite substantial progress in interventional cardiology, pharmacotherapy, and mechanical circulatory support (MCS), the prognosis of CS remains poor, with early mortality rates consistently ranging from 30% to 50%, depending on the underlying aetiology [2,3].

Although AMI remains the most common cause of CS, an increasing proportion of patients present with non-AMI CS, including cases secondary to progressive myocardial inflammation and HF [4,5]. These non-AMI aetiologies are increasingly recognised as distinct clinical entities with different pathophysiological pathways, often requiring individual diagnostic and therapeutic approaches [6]. Yet, they remain underrepresented in major clinical trials and consensus guidelines, which have traditionally focused on AMI-related CS.

The recent literature has emphasised the importance of stratifying CS patients based on the underlying cause to improve outcomes through personalised treatment strategies [4,7,8,9]. However, robust comparative data on the clinical characteristics and prognosis of CS secondary to the progression of HF—referred to here as PHF-CS—versus other forms of CS remain limited. A better understanding of these subgroups is crucial to support risk stratification, guide clinical decision-making, and inform the use of advanced therapies such as MCS.

## 2. Objectives

The primary objective of this study is to compare the clinical characteristics, laboratory parameters, in-hospital management, and outcomes of patients with CS due to the progression of HF (PHF-CS) versus those with cardiogenic shock from other causes (non-PHF-CS). Additionally, we aim to identify factors that may influence survival and therapeutic response in the PHF subgroup, with the goal of supporting a more tailored and evidence-based approach to the management of these patients.

## 3. Materials and Methods

### 3.1. General Characteristics of the Performed Study

This retrospective observational study included patients admitted with CS to a tertiary care hospital between 2 January 2021 and 3 April 2024. All patients received treatment in accordance with standard institutional protocols. Relevant clinical data were collected from medical records and entered into a dedicated research database. The study was conducted in accordance with the Declaration of Helsinki and approved by the Ethics Committee of the Wroclaw Medical University (consent no. ID KB-567/2024). The study was a subanalysis of The Lower Silesia Cardiogenic Shock Initiative, registered on ClinicalTrials.gov under the identifier NCT05465200. Due to the retrospective design, the requirement for informed consent was waived.

### 3.2. Eligibility Criteria

Patients were eligible for inclusion if they were diagnosed with CS during hospitalisation. Exclusion criteria were as follows: (1) patients without the return of spontaneous circulation who were considered for extra-corporeal cardiopulmonary resuscitation, (2) primary septic shock, and (3) postcardiotomy shock. The cohort was subsequently divided into two groups:

(1) PHF group—patients with CS resulting from progression of HF or progressive myocarditis.
(2) non-PHF group—patients with CS of other aetiologies, including acute coronary syndromes (ACS), pulmonary embolism, or other causes.

### 3.3. Clinical and Laboratory Data Collection

The collected clinical data included demographic characteristics, body mass index (BMI), comorbidities, history of myocardial infarction (MI), prior percutaneous coronary interventions (PCI), coronary artery bypass grafting (CABG), and valve procedures. Additionally, risk factors such as smoking and alcohol consumption were recorded. The laboratory data were comprised of complete blood count, *N*-terminal pro–B-type natriuretic peptide (NT-proBNP), troponin T, D-dimers, bilirubin, lactate, pH, inflammatory markers, and renal function parameters. Missing data are reported in the relevant tables.

### 3.4. In-Hospital Course and Interventions

Hospital-related parameters included duration of hospitalisation, use of mechanical ventilation (MV), renal replacement therapy (RRT), coronary revascularisation procedures, occurrence of cerebral events, administration of antibiotics, and left ventricular ejection fraction (EF) at admission and discharge. Moreover, the use of MCS and types of devices implemented (e.g., intra-aortic balloon pump (IABP), Impella, extracorporeal membrane oxygenation (ECMO), left ventricular assist device (LVAD)) were documented.

### 3.5. Statistical Analysis

Continuous variables were assessed for normality using the Shapiro–Wilk test. Normally distributed variables are presented as mean ± standard deviation (SD), while non-normally distributed variables are expressed as medians with interquartile ranges (IQR). Comparisons between groups were performed using the Student’s *t*-test or Mann–Whitney U test, as appropriate. Categorical variables are presented as absolute values and percentages; intergroup differences were assessed using Pearson’s chi-square test. For variables with small and expected events (<5) the Fisher exact test was utilized. A *p*-value of <0.05 was considered statistically significant. Statistical analysis was performed using STATISTICA software, version 13.3 (StatSoft, licensed to Wroclaw Medical University).

## 4. Results

### 4.1. General Characteristics

The study included 280 patients with a median age of 67 (60–76) years old, 197 (70%) were male. The PHF group consisted of 84 (30%) patients: 82 with progression of HF and 2 with inflammation of cardiac muscle. The non-PHF group consisted of 196 (70%) patients: 164 (59%) with AMI and its complications, 14 (5%) with pulmonary embolism, and 20 (7%) with other causes of CS. According to the clinical characteristics, the PHF group was significantly younger, 65 years (49–74), than the non-PHF group, 69 years (63–78), *p* < 0.01 and included more male patients (81% vs. 66%), *p* = 0.01. In the PHF group, more patients suffered from chronic kidney disease (CKD) (30% vs. 15%), *p* < 0.01, and significant valve disease (30% vs. 13%), *p* < 0.01. Full clinical characteristics are present in Table 1.

### 4.2. Laboratory Tests

Laboratory parameters revealed a higher level of creatinine among patients with diabetes mellitus (1.48 [1.12–2.16] vs. 1.32 [0.94–1.78], *p* = 0.02) and a significantly lower glomerular filtration rate (48 [30–69] vs. 54 [36–78], *p* = 0.03). In terms of baseline laboratory parameters, PHF-group patients had significantly lower levels of white blood cells (WBC) (9.4 [6.9–16.4] vs. 13.3 [10.4–17.6], *p* < 0.01), and troponin T levels (188 [61–1392] vs. 10,921 [809–45,792], *p* = 0.00). Full laboratory characteristics are presented in Table 2.

### 4.3. Hospitalisation Characteristics

The study groups significantly differed in their Society for Cardiovascular Angiography & Interventions (SCAI) profiles with less advanced stages in PHF patients: 48% vs. 31%; 38% vs. 45%; 14% vs. 23%, respectively, C, D, and E class, *p* = 0.02, see Table 3.

There was a significant difference in in-hospital mortality, which was lower in the PHF group (52% vs. 65%, *p* = 0.04). There was no difference in usage of MCS, but there were significant differences in the type of MCS, *p* < 0.01. There was lower use of IABP (15% vs. 43%) and Impella CP (9% vs. 23%) in the PHF group, but higher use of Impella + ECMO (18% vs. 11%), HeartMate 3 (18% vs. 0%), and Impella 5.5 after the upgrade from Impella CP (9% vs. 4%). Moreover, the PHF group had a lower EF at admission (26 [18–35] vs. 35 [25–45], *p* < 0.01) and at discharge (20 [17–42] vs. 38 [26–52], *p* = 0.03). Additionally, the patients from the PHF group underwent less coronary revascularisation (15% vs. 70%), *p* = 0.00. Full hospital characteristics are detailed in Table 4 and Table 5.

## 5. Discussion

To our knowledge, this is the first study to directly compare patients with CS resulting from PHF with those whose CS originated from other aetiologies (non-PHF). Our analysis revealed that the PHF cohort was significantly younger and included a higher proportion of male patients compared to the non-PHF group. Similar demographic trends have been observed in prior studies, suggesting that younger men may be more susceptible to progressive HF and its complications [10]. This may be partially explained by the protective cardiovascular effects of oestrogens, particularly in relation to endothelial function and hypoxia tolerance, although the specific role of sex hormones in CS pathophysiology remains unclear [10]. Despite these demographic associations, the literature provides inconsistent findings regarding sex- and age-based differences in outcomes or treatment strategies in CS [10,11,12,13,14]. Patients with myocarditis were included in the PHF group due to the development of HF following myocardial inflammation. Viral infection remains the most common cause of myocarditis, and its clinical course can vary widely—from asymptomatic cases to fulminant HF or sudden cardiac death [15,16,17]. Notably, myocarditis-related CS has been reported in the context of infections such as COVID-19 or influenza [18,19,20].

In terms of comorbidities, the PHF group demonstrated a higher prevalence of CKD and significant valvular disease. These observations are in line with the current understanding of the interconnected pathophysiological mechanisms underlying cardiorenal syndrome and valvular dysfunction, all of which contribute to HF progression and poor prognosis [21,22,23].

An important clinical observation was that patients in the PHF group presented with less advanced stages of CS according to the SCAI classification. This may reflect the typically gradual decline in cardiac function in PHF-CS, allowing for partial compensation and physiological adaptation. In contrast, ACS in the non-PHF group often lead to abrupt hemodynamic deterioration, which may be mirrored by markedly elevated troponin levels in that cohort. Interestingly, the significantly lower WBC count observed in PHF patients may be attributable to chronic low-grade inflammation and immune exhaustion seen in long-standing HF, rather than the acute inflammatory response elicited by myocardial necrosis in ACS [24]. In addition, chronic HF is known to affect bone marrow function and hematopoietic reserve, which could further contribute to reduced WBC responsiveness in this population [25]. Previous studies have also indicated that elevated WBC levels are independently associated with increased 30-day mortality in both ACS and CS populations [26]. Finally, the association between lower SCAI stage and reduced in-hospital mortality in the PHF group may highlight the prognostic utility of SCAI staging in differentiating shock severity [27]. This suggests that the less acute hemodynamic compromise, as indicated by earlier SCAI stages, may contribute to the improved short-term outcomes in the PHF group. The more progressive nature of decompensation in PHF-CS may allow for earlier recognition and intervention, potentially mitigating mortality risk.

Another potential contributor to the lower in-hospital mortality in PHF-CS patients may be the differences in the use of MCS. More advanced therapies, such as combined Impella and ECMO (ECMELLA), upgrades from Impella CP to Impella 5.5, and LVAD implantation, were more frequently applied in the PHF group. In contrast, the non-PHF group more often received initial support with IABP or standalone Impella CP. This difference may be explained by the significantly lower left ventricular EF at baseline and discharge in PHF patients, necessitating more intensive support. These findings are consistent with existing evidence suggesting that advanced MCS options such as Impella 5.5 and LVADs may improve outcomes in patients with refractory HF [28,29,30]. Furthermore, the use of ECMELLA has been associated with enhanced hemodynamic stabilisation and survival in selected high-risk patients [30,31].

The notably lower rate of coronary revascularisation in the PHF group also warrants consideration. This may be due to the non-ischemic aetiology of shock in these patients, where advanced MCS is prioritized over urgent revascularisation. This management strategy aligns with contemporary guidelines and may reflect more personalised therapeutic approaches in this subgroup [32].

Taken together, the differences in clinical profiles, inflammatory responses, therapeutic approaches, and outcomes between PHF and non-PHF patients with CS underscore the importance of individualised, aetiology-based management strategies. Future prospective studies should focus on identifying predictors of therapeutic response, including biomarkers, imaging parameters, and hemodynamic indices, to optimize patient selection for specific interventions.

This study has several inherent limitations due to its retrospective and single-centre design. The lack of standardised protocols for treatment decisions, which were made at the discretion of individual clinicians, may have introduced variability in clinical management. Additionally, the relatively limited sample size, particularly within the PHF group, affects the generalizability of our findings. These factors emphasise the need for larger, prospective, multicentre studies to validate and expand upon our results, as they provide new insights into the heterogeneity of cardiogenic shock but should be interpreted with caution.

## 6. Conclusions

CS secondary to PHF represents a distinct clinical entity with specific demographic, clinical, and laboratory characteristics compared to other forms of CS. Patients in the PHF group were younger, had a higher burden of comorbidities, and showed different biomarker profiles. Despite the need for more advanced MCS, these patients exhibited lower in-hospital mortality, suggesting a distinct pathophysiology and potentially greater benefit from intensive, individualised treatment strategies. These results highlight the need for prospective studies to validate these findings and guide the development of tailored therapeutic approaches in this growing subgroup of CS patients. These findings may inform clinical decision-making by encouraging the tailoring of mechanical circulatory support strategies to the underlying cause of CS, thereby enhancing therapeutic efficacy and improving outcomes.

## Figures and Tables

**Table 1:** Clinical characteristics of the PHF and non-PHF groups.

Parameter	All Patients (*n* = 280)	Valid *n*	Group: PHF Count (*n* = 84)	Group: Non-PHF Count (*n* = 196)	*p*-Value
Age (IQR)	69 (60–76)	280	65 (49–74)	69 (63–78)	<0.01
Sex, male (%)	197 (70)	280	68 (81)	129 (66)	0.01
BMI kg/m^2^ (IQR)	26.8 (24–29.4)	120	27.5 (23.5–29.4)	26.4 (24.2–29.4)	0.87
Body mass, kg (IQR)	80 (69–86)	134	81 (70–95)	76 (68–85)	0.14
Arterial hypertension, *n* (%)	166 (59)	280	44 (52)	122 (62)	0.12
DM, *n* (%)	99 (35)	280	33 (39)	66 (34)	0.37
CKD, *n* (%)	55 (20)	280	25 (30)	30 (15)	<0.01
Hyperlipidaemia, *n* (%)	65 (23)	280	26 (31)	39 (20)	0.04
Active CA, *n* (%)	14 (5)	280	2 (2)	12 (6)	0.15
MI in past, *n* (%)	79 (28)	280	24 (29)	55 (28)	0.93
PCI in past, *n* (%)	74 (26)	280	26 (31)	48 (24)	0.26
CABG in past, *n* (%)	15 (5)	280	7 (8)	8 (4)	0.15
Significant Valve Disease, *n* (%)	50 (18)	280	25 (30)	25 (13)	<0.01
Valve procedure, *n* (%)	22 (8)	280	9 (11)	13 (7)	0.24
PAD, *n* (%)	36 (13)	280	8 (10)	28 (14)	0.28
CIS, *n* (%)	19 (7)	280	7 (8)	12 (6)	0.50
CHS, *n* (%)	1 (0)	280	0 (0)	1 (1)	0.70
Alcohol Addiction, *n* (%)	31 (11)	280	12 (14)	19 (10)	0.26
Nicotinism in the past/now, *n* (%)	106 (38)	280	30 (36)	76 (39)	0.63

BMI—Body Mass Index, DM—Diabetes Mellitus, CKD—Chronic Kidney Disease, CA—Carcinoma, MI—Myocardial Infarction, PCI—Percutaneous Coronary Interventions, CABG—Coronary Artery Bypass Grafting, PAD—Peripheral Arterial Disease, CIS—Cerebral Ischemic Stroke, and CHS—Cerebral Haemorrhagic Stroke.

**Table 2:** Baseline laboratory characteristics of the PHF and non-PHF groups.

Parameter	All Patients (*n* = 280)	Valid *n*	Group: PHF Count (*n* = 84)	Group: Non-PHF Count (*n* = 196)	*p*-Value
Hb g/dL (SD)	12.1 (2.16)	258	12.3 (2.2)	12 (2.2)	0.31
Ht % (SD)	36.7 (6.3)	258	37.5 (6.4)	36.4 (6.2)	0.17
PLT × 10^9^/L (IQR)	205.5 (148–269)	258	183 (130–299)	208 (163–260)	0.36
WBC × 10^9^/L (IQR)	12.2 (9–17.4)	258	9.4 (6.9–16.4)	13.3 (10.4–17.6)	<0.01
NT-proBNP pg/mL (IQR)	7063 (2119–17,249)	168	7883 (2881–15,390)	6796 (1796–20,787)	0.61
CRP mg/L (IQR)	28 (7.4–86.3)	216	21.9 (8.2–58.3)	32.4 (5.7–97.4)	0.47
Procalcitonin ng/mL (IQR)	0.68 (0.1–2.8)	69	0.2 (0.05–1)	0.83 (0.1–3.18)	0.15
Tn μg/L (IQR)	3587 (189–34,438)	187	188 (61–1392)	10,921 (809–45,792)	<0.01
Creatinine mg/dL (IQR)	1.41 (1.01–1.9)	234	1.42 (1.13–1.94)	1.33 (0.94–1.9)	0.23
GFR mL/min/1.73 m^2^ (IQR)	52 (33–73)	230	54 (32–72)	52 (34–73)	0.93
Bilirubin mg/dL (IQR)	1 (0.6–1.7)	139	1.3 (0.6–2.3)	0.85 (0.6–1.6)	0.07
D-Dimers µg/L (IQR)	4.04 (1.46–17.21)	98	2.36 (1.32–14.26)	5.05 (1.74–19.56)	0.46
Lactate mmol/L (IQR)	3 (1.5–6)	202	2.5 (1.3–5.4)	3.2 (1.6–6.1)	0.19

Hb—Haemoglobin, Ht—Haematocrit, PLT—Platelet Count, WBC—White Blood Cells, NT-proBNP—*N*-Terminal Pro–B-Type Natriuretic Peptide, CRP—C-reactive protein, Tn—Troponin, GFR—Glomerular filtration rate, and pH—Potential of Hydrogen.

**Table 3:** SCAI classification of patients.

Parameter	All Patients (*n* = 280)	Group: PHF Count (*n* = 84)	Group: Non-PHF Count (*n* = 196)	*p*-Value
C, *n* (%)	101 (36)	40 (48)	61 (31)	0.02
D, *n* (%)	121 (43)	32 (38)	89 (45)	
E, *n* (%)	58 (21)	12 (14)	46 (23)	

SCAI—Society for Cardiovascular Angiography & Interventions.

**Table 4:** Hospital characteristics of the PHF and non-PHF groups.

Parameter	All Patients (*n* = 280)	Valid *n*	Group: PHF Count (*n* = 84)	Group: Non-PHF Count (*n* = 196)	*p*-Value
Hospitalisation time, days (IQR)	12 (3–24)	280	16 (8–30)	10 (2–22)	<0.01
MV, *n* (%)	134 (48)	280	33 (39)	101 (52)	0.06
RRT, *n* (%)	56 (20)	280	15 (18)	41 (21)	0.56
MCS, *n* (%)	115 (41)	280	33 (39)	82 (42)	0.69
Coronary revascularisation, *n* (%)	50 (18)	280	13 (15)	137 (70)	<0.01
EF at admission, % (IQR)	31 (22–45)	231	26 (18–35)	35 (25–45)	<0.01
EF at discharge, % (IQR)	33 (20–49)	84	20 (17–42)	38 (26–52)	0.01
Mortality, *n* (%)	172 (61)	280	44 (52)	128 (65)	0.04

MV—Mechanical Ventilation, RRT—Renal Replacement Therapy, MCS—Mechanical Circulatory Support, and EF—Ejection Fraction.

**Table 5:** The use types of MCS therapy.

Parameter	All Patients (*n* = 115)	Group: PHF Count (*n* = 33)	Group: Non-PHF Count (*n* = 82)	*p*-Value
IABP, *n* (%)	40 (35)	5 (15)	35 (43)	<0.01
Impella CP, *n* (%)	22 (19)	3 (9)	19 (23)	
Impella 5.5, *n* (%)	8 (7)	5 (15)	3 (4)	
Impella + ECMO, *n* (%)	15 (13)	6 (18)	9 (11)	
HeartMate 3, *n* (%)	6 (5)	6 (18)	0 (0)	
ECMO + IABP, *n* (%)	10 (9)	3 (9)	7 (9)	
Uprage IABP to Impella CP, *n* (%)	4 (3)	1 (3)	3 (4)	
Uprage IABP to Impella 5.5, *n* (%)	4 (3)	1 (3)	3 (4)	
Upgrade Impella CP to Impella 5.5, *n* (%)	6 (5)	3 (9)	3 (4)	

IABP—Intra-Aortic Balloon Pump, CP—Cardiac power, and ECMO—Extracorporeal membrane oxygenation.

## Data Availability

Data are contained within the article.
